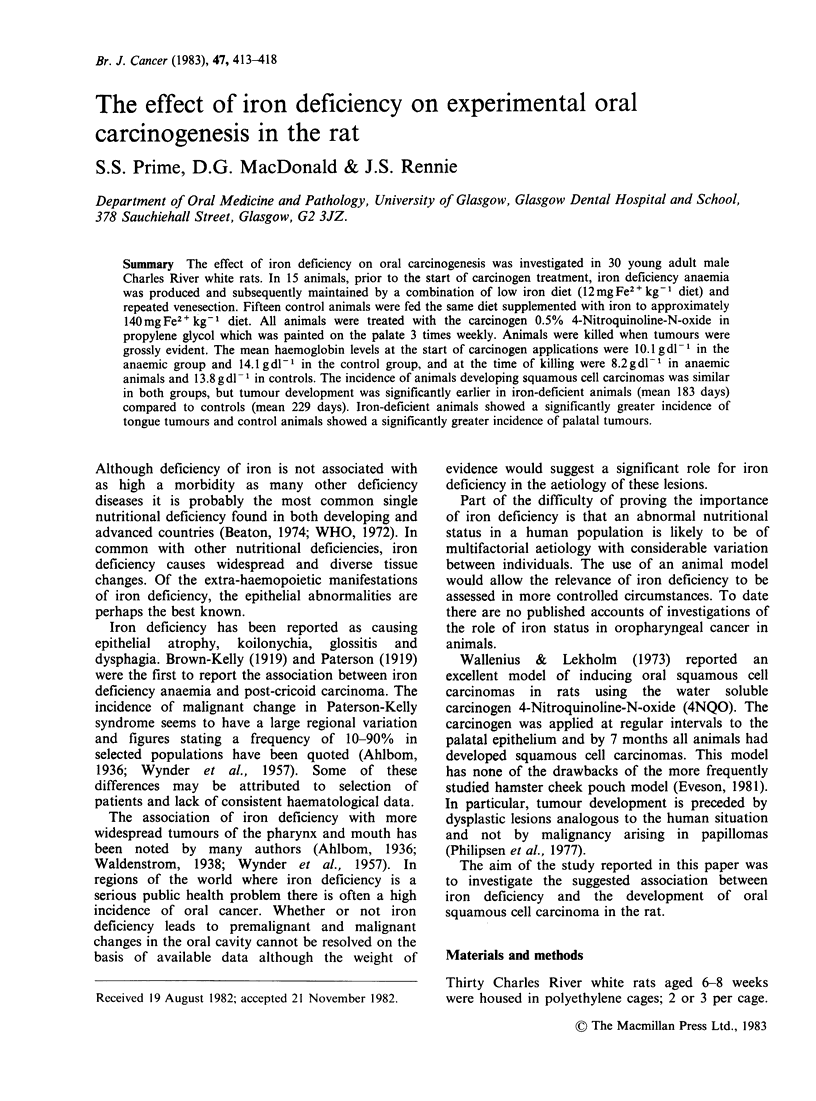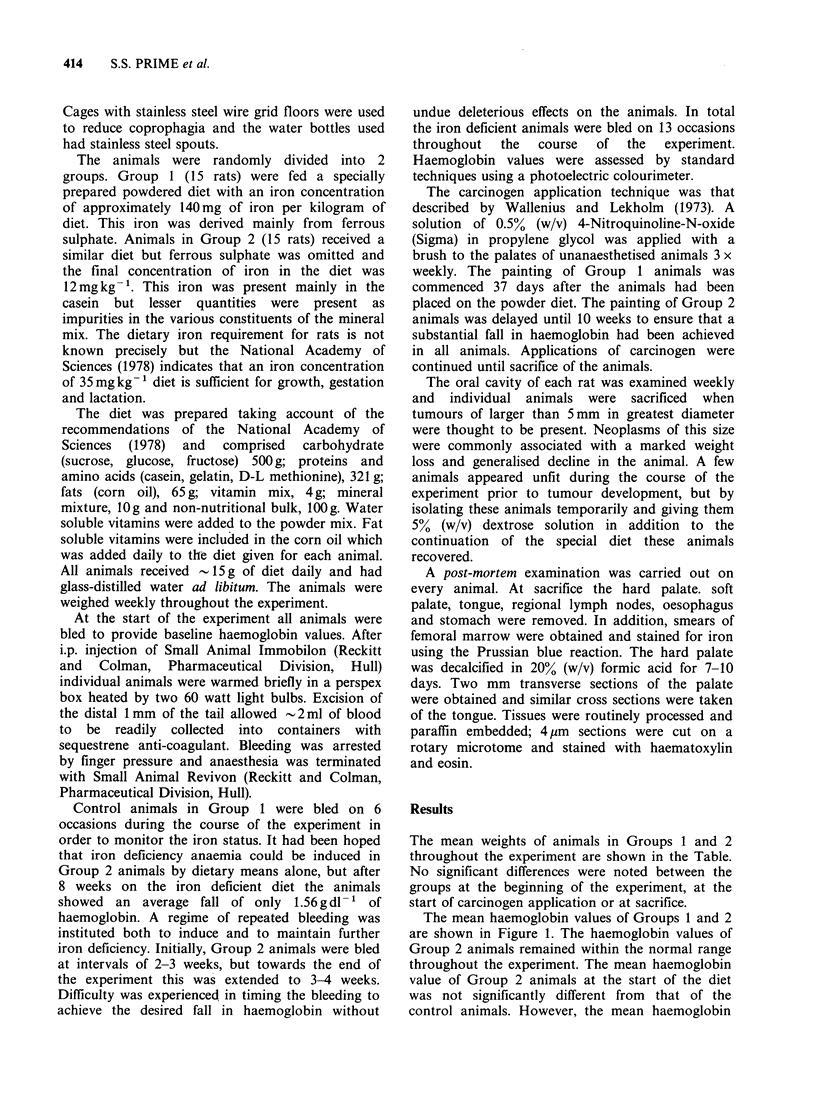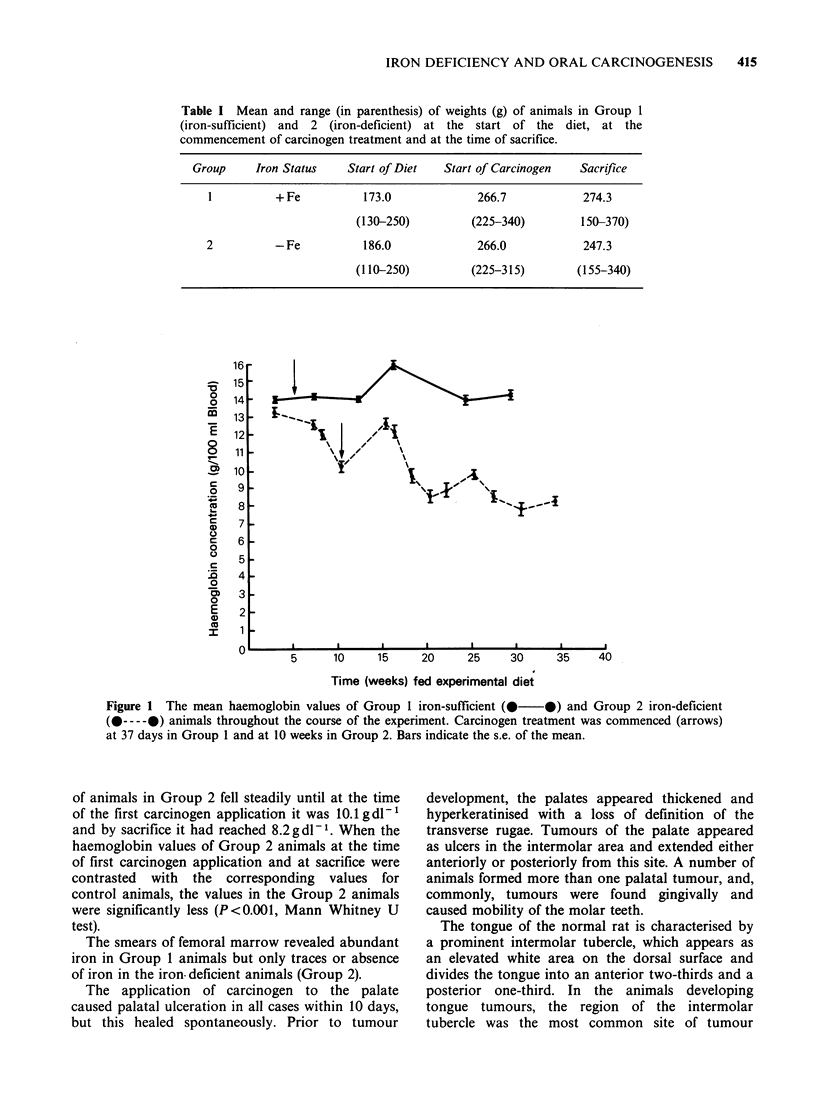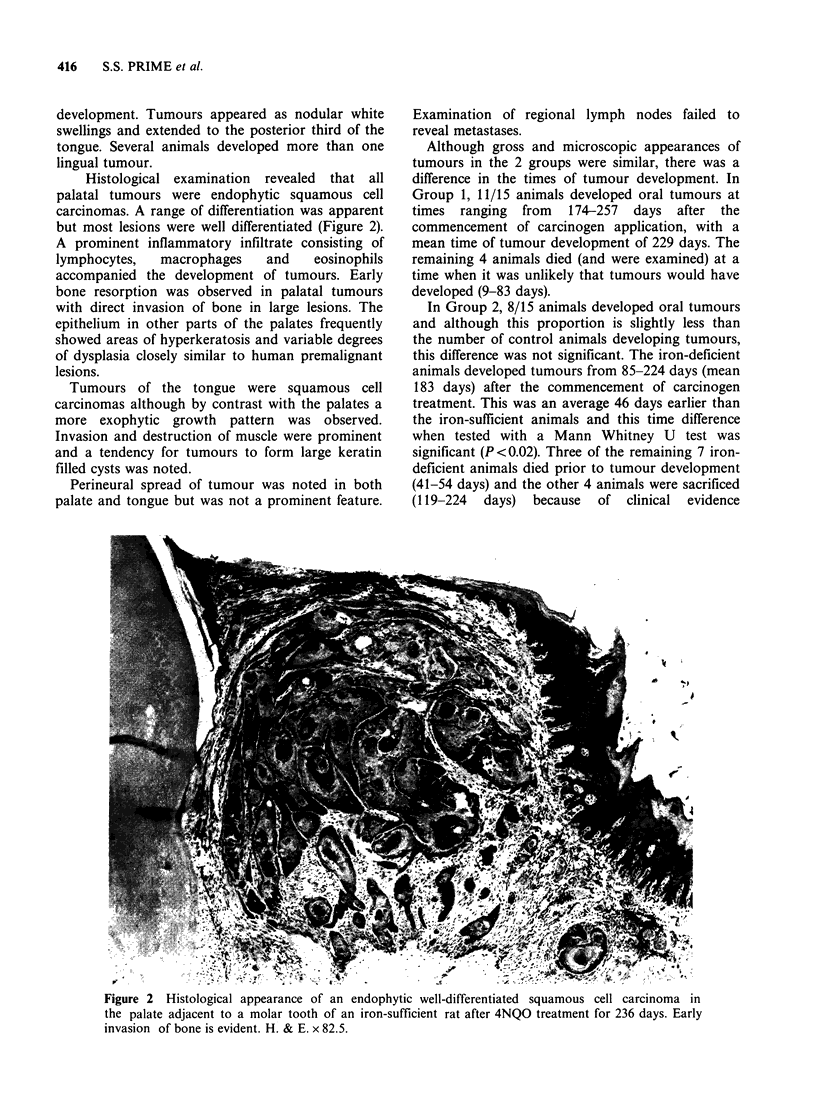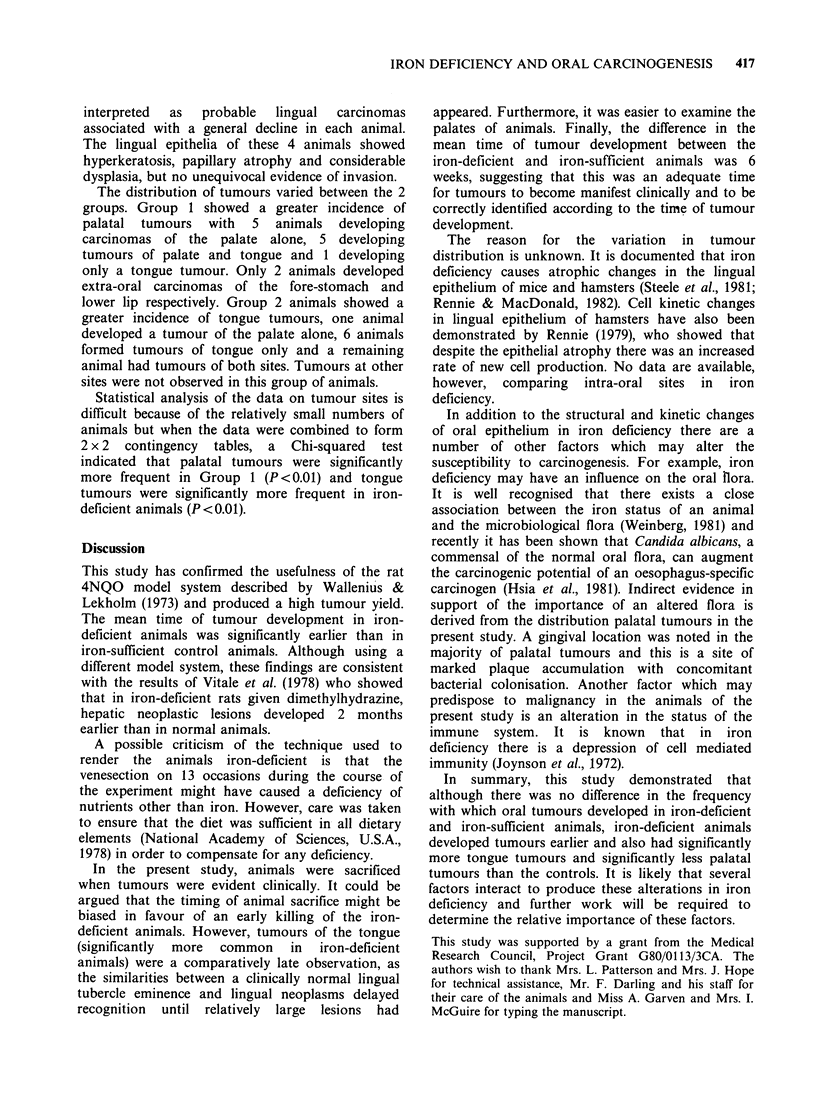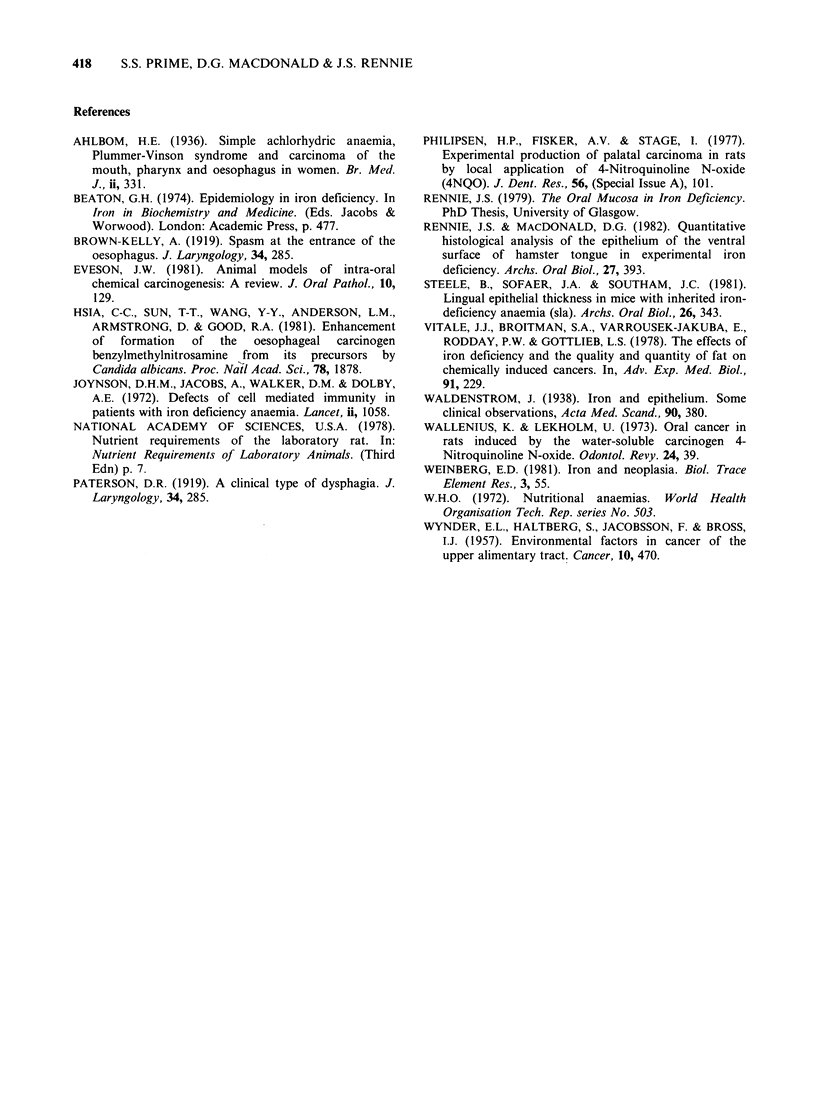# The effect of iron deficiency on experimental oral carcinogenesis in the rat.

**DOI:** 10.1038/bjc.1983.62

**Published:** 1983-03

**Authors:** S. S. Prime, D. G. MacDonald, J. S. Rennie

## Abstract

**Images:**


*Br. J. Cancer* (1983), **47**, 413–418

### The effect of iron deficiency on experimental oral carcinogenesis in the rat

S.S. Prime, D.G. MacDonald & J.S. Rennie

*Department of Oral Medicine and Pathology, University of Glasgow, Glasgow Dental Hospital and School, 378 Sauchiehall Street, Glasgow, G2 3JZ.*

**Summary** The effect of iron deficiency on oral carcinogenesis was investigated in 30 young adult male Charles River white rats. In 15 animals, prior to the start of carcinogen treatment, iron deficiency anaemia was produced and subsequently maintained by a combination of low iron diet ($12\,mg\,Fe^{2+}\,kg^{-1}$ diet) and repeated venesection. Fifteen control animals were fed the same diet supplemented with iron to approximately $140\,mg\,Fe^{2+}\,kg^{-1}$ diet. All animals were treated with the carcinogen 0.5% 4-Nitroquinoline-N-oxide in propylene glycol which was painted on the palate 3 times weekly. Animals were killed when tumours were grossly evident. The mean haemoglobin levels at the start of carcinogen applications were $10.1\,g\,dl^{-1}$ in the anaemic group and $14.1\,g\,dl^{-1}$ in the control group, and at the time of killing were $8.2\,g\,dl^{-1}$ in anaemic animals and $13.8\,g\,dl^{-1}$ in controls. The incidence of animals developing squamous cell carcinomas was similar in both groups, but tumour development was significantly earlier in iron-deficient animals (mean 183 days) compared to controls (mean 229 days). Iron-deficient animals showed a significantly greater incidence of tongue tumours and control animals showed a significantly greater incidence of palatal tumours.

Although deficiency of iron is not associated with as high a morbidity as many other deficiency diseases it is probably the most common single nutritional deficiency found in both developing and advanced countries (Beaton, 1974; WHO, 1972). In common with other nutritional deficiencies, iron deficiency causes widespread and diverse tissue changes. Of the extra-haemopoietic manifestations of iron deficiency, the epithelial abnormalities are perhaps the best known.

Iron deficiency has been reported as causing epithelial atrophy, koilonychia, glossitis and dysphagia. Brown-Kelly (1919) and Paterson (1919) were the first to report the association between iron deficiency anaemia and post-cricoid carcinoma. The incidence of malignant change in Paterson-Kelly syndrome seems to have a large regional variation and figures stating a frequency of 10–90% in selected populations have been quoted (Ahlbom, 1936; Wynder *et al.*, 1957). Some of these differences may be attributed to selection of patients and lack of consistent haematological data.

The association of iron deficiency with more widespread tumours of the pharynx and mouth has been noted by many authors (Ahlbom, 1936; Waldenstrom, 1938; Wynder *et al.*, 1957). In regions of the world where iron deficiency is a serious public health problem there is often a high incidence of oral cancer. Whether or not iron deficiency leads to premalignant and malignant changes in the oral cavity cannot be resolved on the basis of available data although the weight of evidence would suggest a significant role for iron deficiency in the aetiology of these lesions.

Part of the difficulty of proving the importance of iron deficiency is that an abnormal nutritional status in a human population is likely to be of multifactorial aetiology with considerable variation between individuals. The use of an animal model would allow the relevance of iron deficiency to be assessed in more controlled circumstances. To date there are no published accounts of investigations of the role of iron status in oropharyngeal cancer in animals.

Wallenius & Lekholm (1973) reported an excellent model of inducing oral squamous cell carcinomas in rats using the water soluble carcinogen 4-Nitroquinoline-N-oxide (4NQO). The carcinogen was applied at regular intervals to the palatal epithelium and by 7 months all animals had developed squamous cell carcinomas. This model has none of the drawbacks of the more frequently studied hamster cheek pouch model (Eveson, 1981). In particular, tumour development is preceded by dysplastic lesions analogous to the human situation and not by malignancy arising in papillomas (Philipsen *et al.*, 1977).

The aim of the study reported in this paper was to investigate the suggested association between iron deficiency and the development of oral squamous cell carcinoma in the rat.

### Materials and methods

Thirty Charles River white rats aged 6–8 weeks were housed in polyethylene cages; 2 or 3 per cage.

Received 19 August 1982; accepted 21 November 1982.

© The Macmillan Press Ltd., 1983

Cages with stainless steel wire grid floors were used to reduce coprophagia and the water bottles used had stainless steel spouts.

The animals were randomly divided into 2 groups. Group 1 (15 rats) were fed a specially prepared powdered diet with an iron concentration of approximately 140 mg of iron per kilogram of diet. This iron was derived mainly from ferrous sulphate. Animals in Group 2 (15 rats) received a similar diet but ferrous sulphate was omitted and the final concentration of iron in the diet was $12\,mg\,kg^{-1}$. This iron was present mainly in the casein but lesser quantities were present as impurities in the various constituents of the mineral mix. The dietary iron requirement for rats is not known precisely but the National Academy of Sciences (1978) indicates that an iron concentration of $35\,mg\,kg^{-1}$ diet is sufficient for growth, gestation and lactation.

The diet was prepared taking account of the recommendations of the National Academy of Sciences (1978) and comprised carbohydrate (sucrose, glucose, fructose) 500 g; proteins and amino acids (casein, gelatin, D-L methionine), 321 g; fats (corn oil), 65 g; vitamin mix, 4 g; mineral mixture, 10 g and non-nutritional bulk, 100 g. Water soluble vitamins were added to the powder mix. Fat soluble vitamins were included in the corn oil which was added daily to the diet given for each animal. All animals received ~15 g of diet daily and had glass-distilled water *ad libitum*. The animals were weighed weekly throughout the experiment.

At the start of the experiment all animals were bled to provide baseline haemoglobin values. After i.p. injection of Small Animal Immobilon (Reckitt and Colman, Pharmaceutical Division, Hull) individual animals were warmed briefly in a perspex box heated by two 60 watt light bulbs. Excision of the distal 1 mm of the tail allowed ~2 ml of blood to be readily collected into containers with sequestrene anti-coagulant. Bleeding was arrested by finger pressure and anaesthesia was terminated with Small Animal Revivon (Reckitt and Colman, Pharmaceutical Division, Hull).

Control animals in Group 1 were bled on 6 occasions during the course of the experiment in order to monitor the iron status. It had been hoped that iron deficiency anaemia could be induced in Group 2 animals by dietary means alone, but after 8 weeks on the iron deficient diet the animals showed an average fall of only $1.56\,g\,dl^{-1}$ of haemoglobin. A regime of repeated bleeding was instituted both to induce and to maintain further iron deficiency. Initially, Group 2 animals were bled at intervals of 2–3 weeks, but towards the end of the experiment this was extended to 3–4 weeks. Difficulty was experienced in timing the bleeding to achieve the desired fall in haemoglobin without undue deleterious effects on the animals. In total the iron deficient animals were bled on 13 occasions throughout the course of the experiment. Haemoglobin values were assessed by standard techniques using a photoelectric colourimeter.

The carcinogen application technique was that described by Wallenius and Lekholm (1973). A solution of 0.5% (w/v) 4-Nitroquinoline-N-oxide (Sigma) in propylene glycol was applied with a brush to the palates of unanaesthetised animals 3 × weekly. The painting of Group 1 animals was commenced 37 days after the animals had been placed on the powder diet. The painting of Group 2 animals was delayed until 10 weeks to ensure that a substantial fall in haemoglobin had been achieved in all animals. Applications of carcinogen were continued until sacrifice of the animals.

The oral cavity of each rat was examined weekly and individual animals were sacrificed when tumours of larger than 5 mm in greatest diameter were thought to be present. Neoplasms of this size were commonly associated with a marked weight loss and generalised decline in the animal. A few animals appeared unfit during the course of the experiment prior to tumour development, but by isolating these animals temporarily and giving them 5% (w/v) dextrose solution in addition to the continuation of the special diet these animals recovered.

A *post-mortem* examination was carried out on every animal. At sacrifice the hard palate. soft palate, tongue, regional lymph nodes, oesophagus and stomach were removed. In addition, smears of femoral marrow were obtained and stained for iron using the Prussian blue reaction. The hard palate was decalcified in 20% (w/v) formic acid for 7–10 days. Two mm transverse sections of the palate were obtained and similar cross sections were taken of the tongue. Tissues were routinely processed and paraffin embedded; 4 $\mu$m sections were cut on a rotary microtome and stained with haematoxylin and eosin.

### Results

The mean weights of animals in Groups 1 and 2 throughout the experiment are shown in the Table. No significant differences were noted between the groups at the beginning of the experiment, at the start of carcinogen application or at sacrifice.

The mean haemoglobin values of Groups 1 and 2 are shown in Figure 1. The haemoglobin values of Group 2 animals remained within the normal range throughout the experiment. The mean haemoglobin value of Group 2 animals at the start of the diet was not significantly different from that of the control animals. However, the mean haemoglobin

**Table I** Mean and range (in parenthesis) of weights (g) of animals in Group 1 (iron-sufficient) and 2 (iron-deficient) at the start of the diet, at the commencement of carcinogen treatment and at the time of sacrifice.

| *Group* | *Iron Status* | *Start of Diet* | *Start of Carcinogen* | *Sacrifice* |
|---|---|---|---|---|
| 1 | +Fe | 173.0 | 266.7 | 274.3 |
| | | (130–250) | (225–340) | 150–370) |
| 2 | −Fe | 186.0 | 266.0 | 247.3 |
| | | (110–250) | (225–315) | (155–340) |

**Figure 1** The mean haemoglobin values of Group 1 iron-sufficient (●——●) and Group 2 iron-deficient (●----●) animals throughout the course of the experiment. Carcinogen treatment was commenced (arrows) at 37 days in Group 1 and at 10 weeks in Group 2. Bars indicate the s.e. of the mean.

of animals in Group 2 fell steadily until at the time of the first carcinogen application it was 10.1 g dl$^{-1}$ and by sacrifice it had reached 8.2 g dl$^{-1}$. When the haemoglobin values of Group 2 animals at the time of first carcinogen application and at sacrifice were contrasted with the corresponding values for control animals, the values in the Group 2 animals were significantly less ($P<0.001$, Mann Whitney U test).

The smears of femoral marrow revealed abundant iron in Group 1 animals but only traces or absence of iron in the iron-deficient animals (Group 2).

The application of carcinogen to the palate caused palatal ulceration in all cases within 10 days, but this healed spontaneously. Prior to tumour development, the palates appeared thickened and hyperkeratinised with a loss of definition of the transverse rugae. Tumours of the palate appeared as ulcers in the intermolar area and extended either anteriorly or posteriorly from this site. A number of animals formed more than one palatal tumour, and, commonly, tumours were found gingivally and caused mobility of the molar teeth.

The tongue of the normal rat is characterised by a prominent intermolar tubercle, which appears as an elevated white area on the dorsal surface and divides the tongue into an anterior two-thirds and a posterior one-third. In the animals developing tongue tumours, the region of the intermolar tubercle was the most common site of tumour development. Tumours appeared as nodular white swellings and extended to the posterior third of the tongue. Several animals developed more than one lingual tumour.

Histological examination revealed that all palatal tumours were endophytic squamous cell carcinomas. A range of differentiation was apparent but most lesions were well differentiated (Figure 2). A prominent inflammatory infiltrate consisting of lymphocytes, macrophages and eosinophils accompanied the development of tumours. Early bone resorption was observed in palatal tumours with direct invasion of bone in large lesions. The epithelium in other parts of the palates frequently showed areas of hyperkeratosis and variable degrees of dysplasia closely similar to human premalignant lesions.

Tumours of the tongue were squamous cell carcinomas although by contrast with the palates a more exophytic growth pattern was observed. Invasion and destruction of muscle were prominent and a tendency for tumours to form large keratin filled cysts was noted.

Perineural spread of tumour was noted in both palate and tongue but was not a prominent feature. Examination of regional lymph nodes failed to reveal metastases.

Although gross and microscopic appearances of tumours in the 2 groups were similar, there was a difference in the times of tumour development. In Group 1, 11/15 animals developed oral tumours at times ranging from 174–257 days after the commencement of carcinogen application, with a mean time of tumour development of 229 days. The remaining 4 animals died (and were examined) at a time when it was unlikely that tumours would have developed (9–83 days).

In Group 2, 8/15 animals developed oral tumours and although this proportion is slightly less than the number of control animals developing tumours, this difference was not significant. The iron-deficient animals developed tumours from 85–224 days (mean 183 days) after the commencement of carcinogen treatment. This was an average 46 days earlier than the iron-sufficient animals and this time difference when tested with a Mann Whitney U test was significant ($P<0.02$). Three of the remaining 7 iron-deficient animals died prior to tumour development (41–54 days) and the other 4 animals were sacrificed (119–224 days) because of clinical evidence

**Figure 2** Histological appearance of an endophytic well-differentiated squamous cell carcinoma in the palate adjacent to a molar tooth of an iron-sufficient rat after 4NQO treatment for 236 days. Early invasion of bone is evident. H. & E. × 82.5.

interpreted as probable lingual carcinomas associated with a general decline in each animal. The lingual epithelia of these 4 animals showed hyperkeratosis, papillary atrophy and considerable dysplasia, but no unequivocal evidence of invasion.

The distribution of tumours varied between the 2 groups. Group 1 showed a greater incidence of palatal tumours with 5 animals developing carcinomas of the palate alone, 5 developing tumours of palate and tongue and 1 developing only a tongue tumour. Only 2 animals developed extra-oral carcinomas of the fore-stomach and lower lip respectively. Group 2 animals showed a greater incidence of tongue tumours, one animal developed a tumour of the palate alone, 6 animals formed tumours of tongue only and a remaining animal had tumours of both sites. Tumours at other sites were not observed in this group of animals.

Statistical analysis of the data on tumour sites is difficult because of the relatively small numbers of animals but when the data were combined to form $2 \times 2$ contingency tables, a Chi-squared test indicated that palatal tumours were significantly more frequent in Group 1 ($P < 0.01$) and tongue tumours were significantly more frequent in iron-deficient animals ($P < 0.01$).

### Discussion

This study has confirmed the usefulness of the rat 4NQO model system described by Wallenius & Lekholm (1973) and produced a high tumour yield. The mean time of tumour development in iron-deficient animals was significantly earlier than in iron-sufficient control animals. Although using a different model system, these findings are consistent with the results of Vitale *et al.* (1978) who showed that in iron-deficient rats given dimethylhydrazine, hepatic neoplastic lesions developed 2 months earlier than in normal animals.

A possible criticism of the technique used to render the animals iron-deficient is that the venesection on 13 occasions during the course of the experiment might have caused a deficiency of nutrients other than iron. However, care was taken to ensure that the diet was sufficient in all dietary elements (National Academy of Sciences, U.S.A., 1978) in order to compensate for any deficiency.

In the present study, animals were sacrificed when tumours were evident clinically. It could be argued that the timing of animal sacrifice might be biased in favour of an early killing of the iron-deficient animals. However, tumours of the tongue (significantly more common in iron-deficient animals) were a comparatively late observation, as the similarities between a clinically normal lingual tubercle eminence and lingual neoplasms delayed recognition until relatively large lesions had appeared. Furthermore, it was easier to examine the palates of animals. Finally, the difference in the mean time of tumour development between the iron-deficient and iron-sufficient animals was 6 weeks, suggesting that this was an adequate time for tumours to become manifest clinically and to be correctly identified according to the time of tumour development.

The reason for the variation in tumour distribution is unknown. It is documented that iron deficiency causes atrophic changes in the lingual epithelium of mice and hamsters (Steele *et al.*, 1981; Rennie & MacDonald, 1982). Cell kinetic changes in lingual epithelium of hamsters have also been demonstrated by Rennie (1979), who showed that despite the epithelial atrophy there was an increased rate of new cell production. No data are available, however, comparing intra-oral sites in iron deficiency.

In addition to the structural and kinetic changes of oral epithelium in iron deficiency there are a number of other factors which may alter the susceptibility to carcinogenesis. For example, iron deficiency may have an influence on the oral flora. It is well recognised that there exists a close association between the iron status of an animal and the microbiological flora (Weinberg, 1981) and recently it has been shown that *Candida albicans*, a commensal of the normal oral flora, can augment the carcinogenic potential of an oesophagus-specific carcinogen (Hsia *et al.*, 1981). Indirect evidence in support of the importance of an altered flora is derived from the distribution palatal tumours in the present study. A gingival location was noted in the majority of palatal tumours and this is a site of marked plaque accumulation with concomitant bacterial colonisation. Another factor which may predispose to malignancy in the animals of the present study is an alteration in the status of the immune system. It is known that in iron deficiency there is a depression of cell mediated immunity (Joynson *et al.*, 1972).

In summary, this study demonstrated that although there was no difference in the frequency with which oral tumours developed in iron-deficient and iron-sufficient animals, iron-deficient animals developed tumours earlier and also had significantly more tongue tumours and significantly less palatal tumours than the controls. It is likely that several factors interact to produce these alterations in iron deficiency and further work will be required to determine the relative importance of these factors.

This study was supported by a grant from the Medical Research Council, Project Grant G80/0113/3CA. The authors wish to thank Mrs. L. Patterson and Mrs. J. Hope for technical assistance, Mr. F. Darling and his staff for their care of the animals and Miss A. Garven and Mrs. I. McGuire for typing the manuscript.

### References

AHLBOM, H.E. (1936). Simple achlorhydric anaemia, Plummer-Vinson syndrome and carcinoma of the mouth, pharynx and oesophagus in women. *Br. Med. J.*, **ii**, 331.

BEATON, G.H. (1974). Epidemiology in iron deficiency. In *Iron in Biochemistry and Medicine*. (Eds. Jacobs & Worwood). London: Academic Press, p. 477.

BROWN-KELLY, A. (1919). Spasm at the entrance of the oesophagus. *J. Laryngology*, **34**, 285.

EVESON, J.W. (1981). Animal models of intra-oral chemical carcinogenesis: A review. *J. Oral Pathol.*, **10**, 129.

HSIA, C-C., SUN, T-T., WANG, Y-Y., ANDERSON, L.M., ARMSTRONG, D. & GOOD, R.A. (1981). Enhancement of formation of the oesophageal carcinogen benzylmethylnitrosamine from its precursors by *Candida albicans*. *Proc. Natl Acad. Sci.*, **78**, 1878.

JOYNSON, D.H.M., JACOBS, A., WALKER, D.M. & DOLBY, A.E. (1972). Defects of cell mediated immunity in patients with iron deficiency anaemia. *Lancet*, **ii**, 1058.

NATIONAL ACADEMY OF SCIENCES, U.S.A. (1978). Nutrient requirements of the laboratory rat. In: *Nutrient Requirements of Laboratory Animals*. (Third Edn) p. 7.

PATERSON, D.R. (1919). A clinical type of dysphagia. *J. Laryngology*, **34**, 285.

PHILIPSEN, H.P., FISKER, A.V. & STAGE, I. (1977). Experimental production of palatal carcinoma in rats by local application of 4-Nitroquinoline N-oxide (4NQO). *J. Dent. Res.*, **56**, (Special Issue A), 101.

RENNIE, J.S. (1979). *The Oral Mucosa in Iron Deficiency*. PhD Thesis, University of Glasgow.

RENNIE, J.S. & MACDONALD, D.G. (1982). Quantitative histological analysis of the epithelium of the ventral surface of hamster tongue in experimental iron deficiency. *Archs. Oral Biol.*, **27**, 393.

STEELE, B., SOFAER, J.A. & SOUTHAM, J.C. (1981). Lingual epithelial thickness in mice with inherited iron-deficiency anaemia (sla). *Archs. Oral Biol.*, **26**, 343.

VITALE, J.J., BROITMAN, S.A., VARROUSEK-JAKUBA, E., RODDAY, P.W. & GOTTLIEB, L.S. (1978). The effects of iron deficiency and the quality and quantity of fat on chemically induced cancers. In, *Adv. Exp. Med. Biol.*, **91**, 229.

WALDENSTROM, J. (1938). Iron and epithelium. Some clinical observations, *Acta Med. Scand.*, **90**, 380.

WALLENIUS, K. & LEKHOLM, U. (1973). Oral cancer in rats induced by the water-soluble carcinogen 4-Nitroquinoline N-oxide. *Odontol. Revy*. **24**, 39.

WEINBERG, E.D. (1981). Iron and neoplasia. *Biol. Trace Element Res.*, **3**, 55.

W.H.O. (1972). Nutritional anaemias. *World Health Organisation Tech. Rep. series No. 503*.

WYNDER, E.L., HALTBERG, S., JACOBSSON, F. & BROSS, I.J. (1957). Environmental factors in cancer of the upper alimentary tract. *Cancer*, **10**, 470.